# OA19 When the indolent turns lethal: transformation of pulmonary MALT lymphoma to diffuse large B-cell lymphoma in primary Sjögren’s disease

**DOI:** 10.1093/rap/rkag113.019

**Published:** 2026-11-03

**Authors:** Bodhibrata Banerjee, Komal Preet, Charanpreet Singh, Amanjit Bal, Sanjay Jain, Varun Dhir

**Affiliations:** Rheumatology Wing, Department of Internal Medicine, Postgraduate Institute of Medical Education and Research, Chandigarh, India; Department of Nuclear Medicine, Postgraduate Institute of Medical Education and Research, Chandigarh, India; Department of Clinical Hematology and Medical Oncology, Postgraduate Institute of Medical Education and Research, Chandigarh, India; Department of Histopathology, Postgraduate Institute of Medical Education and Research, Chandigarh, India; Rheumatology Wing, Department of Internal Medicine, Postgraduate Institute of Medical Education and Research, Chandigarh, India; Rheumatology Wing, Department of Internal Medicine, Postgraduate Institute of Medical Education and Research, Chandigarh, India

## Abstract

**Introduction:**

Primary Sjögren’s disease (SjD) carries the highest lymphoma risk of any autoimmune disease, affecting roughly 5–10% of patients. The commonest subtype is indolent mucosa-associated lymphoid tissue (MALT) lymphoma (76%), usually arising in the salivary glands or lungs and generally carries an excellent prognosis. A minority, however, undergo histological transformation to more aggresive diffuse large B-cell lymphoma (DLBCL) — a shift that abruptly worsens outcome. We describe a woman with long-standing SjD whose pulmonary MALT lymphoma transformed to DLBCL and pursued a rapidly fatal course.

**Case description:**

A 36-year-old woman had a 13-year history of primary Sjögren’s disease, first presenting with oral sicca, parotid enlargement and polyarthritis, with ANA 3+ speckled and anti-Ro positivity. Five years into her illness she developed renal failure, presumed tubulointerstitial nephritis, which responded to monthly intravenous cyclophosphamide pulses followed by maintenance azathioprine, achieving remission.

She now presented with six months of progressive dyspnoea and dry cough, with scattered crepitations. CT of the thorax showed multiple nodular opacities, several cavitating, and FDG-PET confirmed multiple hypermetabolic nodules. PET-guided biopsy demonstrated extranodal marginal zone (MALT) lymphoma of lung—CD79a-positive small B lymphocytes with MUM1-positive plasma cells and lymphoepithelial lesions. Six cycles of R-CHOP chemotherapy (rituximab, cyclophosphamide, hydroxydaunorubicin, oncovin, prednisone) produced a good clinical and radiological response.

Her course then changed. Transferred to haematology with worsening respiratory distress, she achieved only a temporary response before relapsing. Repeat FDG-PET revealed new avid lesions in the lungs and adrenal glands. Guided biopsy now showed DLBCL (NOS), consistent with histological transformation of her indolent MALT lymphoma. Second-line GDP chemotherapy (gemcitabine, dexamethasone, cisplatin) achieved only a partial response. Two days before death she was admitted at the emergency department with acute myocarditis of uncertain aetiology (disease-related versus infective) and received cyclophosphamide. Despite maximal supportive care, she died.

**Discussion:**

Lung involvement in primary Sjögren’s disease is diverse, spanning airway disease, interstitial pneumonia, amyloidosis, granulomatous disease and lymphoproliferation. When nodules—particularly multiple, cavitating or “pseudo-cavitating” — dominate, lymphoma must be excluded: in one series of SjD patients with pulmonary nodules, non-Hodgkin lymphoma (39%) and MALT lymphoma (37%) were the leading causes, and SjD is the commonest condition underlying pulmonary MALT lymphoma. Tissue diagnosis is essential; imaging alone cannot separate infection from malignancy.

Risk factors for development of lymphoma in SjD are: persistent parotid enlargement, lymphadenopathy, Raynaud’s, anti-Ro/La, rheumatoid factor, monoclonal gammopathy, cryoglobulinaemia, low C4 and high systemic (ESSDAI) activity. Our patient carried a high-risk phenotype from the outset—persistent parotid enlargement, anti-Ro positivity and extraglandular (renal) disease reflecting high systemic activity. MALT lymphoma is usually indolent, with >90% survival. The pivotal adverse event is histological transformation to DLBCL, which accounts for a minority of SjD lymphomas but drives most lymphoma-related deaths, with events clustering within three years. Her abrupt deterioration, new adrenal disease and salvage-refractory course exemplify this trajectory.

Her terminal myocarditis underscores a further dilemma: in a heavily immunosuppressed, multiply-relapsed patient, cardiac inflammation may reflect lymphomatous infiltration, opportunistic infection or anthracycline-related injury—distinctions difficult at the bedside yet therapeutically divergent. The case argues for early, tissue-based recognition of transformation (prompted by rapid clinical change or new FDG-avid extranodal sites), close rheumatology–haematology co-management, and humility about prognosis once indolent disease turns aggressive.

**Key learning points:**

• First, In any SjD patient with persistent or multiple lung nodules—especially cavitating or pseudo—cavitating–actively exclude lymphoma. MALT lymphoma is the leading neoplastic cause and requires a tissue diagnosis, not imaging alone.

• Second, recognise the high-risk SjD phenotype early: persistent parotid enlargement, lymphadenopathy, Raynaud’s, anti-Ro/La, rheumatoid factor, monoclonal gammopathy, cryoglobulinaemia, low C4 and high systemic (ESSDAI) activity are linked to lymphomagenesis. These patients warrant closer surveillance.

• Third, MALT lymphoma is usually indolent, but histological transformation to DLBCL is the decisive lethal event. Suspect it when the course changes abruptly or new FDG-avid extranodal sites appear (here, adrenal) — and re-biopsy rather than assume indolent relapse.

• Fourth, transformed/SjD-associated DLBCL carries a poor prognosis, with adverse events clustering within three years and frequent salvage failure. Involve haemato-oncology early and manage expectations.

• Fifth, new cardiac symptoms in a heavily treated lymphoma patient are a diagnostic emergency—distinguish lymphomatous infiltration, opportunistic infection and anthracycline cardiotoxicity (troponin, echocardiography, cardiac MRI with/without endomyocardial biopsy) before committing to empirical immunosuppression.

• Finally, lymphoma risk—and its transformation—persists across the entire SjD disease course; lifelong rheumatology–haematology co-management is essential.

